# Curcumin Induces Cell Death in Esophageal Cancer Cells through Modulating Notch Signaling

**DOI:** 10.1371/journal.pone.0030590

**Published:** 2012-02-17

**Authors:** Dharmalingam Subramaniam, Sivapriya Ponnurangam, Prabhu Ramamoorthy, David Standing, Richard J. Battafarano, Shrikant Anant, Prateek Sharma

**Affiliations:** 1 Department of Molecular and Integrative Physiology, University of Kansas Medical Center, Kansas City, Kansas, United States of America; 2 Division of Gastroenterology and Hepatology, University of Kansas Medical Center, Kansas City, Kansas, United States of America; 3 The University of Kansas Cancer Center, University of Kansas Medical Center, Kansas City, Kansas, United States of America; 4 Department of Surgery, University of Maryland School of Medicine, Baltimore, Maryland, United States of America; Roswell Park Cancer Institute, United States of America

## Abstract

**Background:**

Curcumin inhibits the growth of esophageal cancer cell lines; however, the mechanism of action is not well understood. It is becoming increasingly clear that aberrant activation of Notch signaling has been associated with the development of esophageal cancer. Here, we have determined that curcumin inhibits esophageal cancer growth via a mechanism mediated through the Notch signaling pathway.

**Methodology/Principal Findings:**

In this study, we show that curcumin treatment resulted in a dose and time dependent inhibition of proliferation and colony formation in esophageal cancer cell lines. Furthermore, curcumin treatment induced apoptosis through caspase 3 activation, confirmed by an increase in the ratio of Bax to Bcl2. Cell cycle analysis demonstrated that curcumin treatment induced cell death and down regulated cyclin D1 levels. Curcumin treatment also resulted in reduced number and size of esophagospheres. Furthermore, curcumin treatment led to reduced Notch-1 activation, expression of Jagged-1 and its downstream target Hes-1. This reduction in Notch-1 activation was determined to be due to the down-regulation of critical components of the γ-secretase complex proteins such as Presenilin 1 and Nicastrin. The combination of a known γ-secretase inhibitor DAPT and curcumin further decreased proliferation and induced apoptosis in esophageal cancer cells. Finally, curcumin treatment down-regulate the expressions of Notch-1 specific microRNAs miR-21 and miR-34a, and upregulated tumor suppressor let-7a miRNA.

**Conclusion/Significance:**

Curcumin is a potent inhibitor of esophageal cancer growth that targets the Notch-1 activating γ-secretase complex proteins. These data suggest that Notch signaling inhibition is a novel mechanism of action for curcumin during therapeutic intervention in esophageal cancers.

## Introduction

Esophageal cancer is the eighth most common incident cancer in the world and sixth in cancer mortality [1]. In the United States, 4–10 in 100,000 persons succumb to the disease per year and the overall incidence of the disease is highest in men over 50 years of age [2]. The American Cancer Society (ACS) estimated that in 2011, 16,980 Americans (13,450 men and 3,530 women) would be diagnosed with esophageal cancer. The ACS also estimated that the majority of these individuals [14, 710 Americans (11,910 men and 2,800 women)] would die of esophageal cancer in 2011 [3]. Esophageal adenocarcinoma, the major form of esophageal cancer in the US, is the most rapidly rising cancer in the western world. It is generally diagnosed at a late stage and has a poor prognosis, with a 5-year survival of less than 10%. Although the current treatment includes chemotherapy, radiation therapy, and, if possible, esophagogastric resection, many patients with esophageal adenocarcinoma experience progression of disease despite such treatment, suggesting that such tumors are resistant to standard therapy.

Because conventional therapies, including surgical resection, chemotherapy, and radiation are often inadequate in treating this disease, new treatment options are critically needed. Despite the emergence of novel targeted agents and the use of various therapeutic combinations, no treatment options are available that are curative in patients with advanced cancer. The magnitude of this problem mandates the need for novel therapeutic agents, specifically the use of agents for chemoprevention. This is most attractive for esophageal adenocarcinoma since a pre-malignant condition -Barrett's esophagus is a well-recognized lesion.

Curcumin, a phyto-polyphenolic pigment derived from turmeric (*Curcuma longa*), has been shown to have multiple anticancer effects, including inhibition of proliferation, induction of apoptosis, inhibition of angiogenesis, and inhibition of DNA topoisomerase II [4]. Curcumin also induces apoptosis-independent death such as autophagy in esophageal cancer cells [5]. Recent studies have demonstrated that curcumin promotes apoptosis, increases chemosensitivity, and inhibits NF-κB in esophageal adenocarcinoma [6] [7].

Notch signaling plays a critical role in the development and homeostasis of tissues by regulating cell-fate decisions, proliferation, differentiation, and apoptosis. Notch signaling pathway is implicated in stem cell self-renewal, cell-fate determination, proliferation, differentiation and apoptosis [8]. Notch signaling is upregulated in esophageal cancers and has been proposed as a therapeutic target for esophageal cancers [9] [10]. Notch signaling is initiated when a notch ligand interacts with a notch transmembrane receptor on a adjacent cells [11] [12]. This process usually initiates the γ-secretase mediated proteolytic release of the Notch intracellular domain (NICD), which, in turn, translocates into the nucleus of the cells [13]. The NICD in the nucleus interacts with the C promoter-binding factor-1 (CBF1) transcriptional cofactor and transactivates the target genes, such as those in the hairy and enhancer of split (Hes) and Hes related with YRPW motif (Hey) families [12] [14]. Recent studies have demonstrated a connection between curcumin, Notch and NF-κB. Notch-1 signaling pathway has been directly shown to be activated by NF-κB in oral cancer cells [15]. Moreover, curcumin-mediated inhibition of Notch-1 activation also led to the downregulation of NF-κB and its target genes, including Bcl-2, cyclin D1, vascular endothelial growth factor (VEGF), and matrix metalloproteinase -9 (MMP-9) in oral squamous cell carcinoma cells [4].

MicroRNAs (miRNAs) are short non-coding RNAs that bind to the 3′ untranslated region (UTR) of cognate messenger RNAs (mRNAs) through fully complementary or imperfect base-pairing repressing the translation or decreasing the stability of the bound mRNAs [16]. Some miRNAs are reported as oncomirs which could function as either oncogenes or tumor suppressors [17]. For example, miR-21 decreases tumor suppressor Pdcd4 expression and promotes invasion, intravasation and metastasis in colorectal cancer [18]. Upregulation of miR-21 is strongly associated with both a high Ki-67 proliferative index and the presence of liver metastasis [19]. miR-21 also regulated PTEN-dependent pathway and affected cell growth, migration and invasion of hepatocellular carcinoma[20]. Recently, we have demonstrated miRNAs as biomarkers for Barrett's esophagus progression. miR-21 is upregulated 12.57-fold in esophageal cancer [21]. Another important miRNA is miR-34, which has been found to participate in the regulation of p53 and Notch pathways consistent with tumor suppressor activity [22]. A recent study demonstrated that there is a cross-talk between miRNA and Notch signaling pathways in tumor development and progression [23]. Moreover, Notch signaling and its regulations are critically important at the level of post-transcriptional and/or translational regulation of genes by miRNAs in glioblastoma [24]. On the other hand, let-7a decreases cell proliferation and migration of glioblastoma and reduces tumor size in xenograft model [25].

In this article, we have determined the effect of curcumin on esophageal cancer cells and mechanism mediated through Notch signaling pathway.

## Materials and Methods

### Cells and Reagents

TE-7, TE-10 are human and ESO-1 is mouse esophageal adenocarcinoma cancer cells that were kindly provided by Dr. Richard J. Battafarano, University of Maryland and grown in RPMI 1640 containing 10% heat inactivated fetal bovine serum (Sigma-Aldrich, St. Louis, MO) and 1% antibiotic-anti-mycotic solution (Mediatech Inc, Manassas, VA) at 37°C in a humidified atmosphere of 5% CO_2_. Curcumin was purchased from LKT Laboratories, St. Paul, MN. N-[N-(3,5-Difluorophenacetyl)-L-alanyl]-S-phenylglycine t-butyl ester (DAPT) was purchased (Sigma-Aldrich, St. Louis, MO).

### Proliferation and Apoptosis assays

To assess proliferation, cells were seeded on to 96 well plates and grow overnight. Then the cells were treated with increasing doses of curcumin in 10% FBS containing RPMI 1640. Analysis of cell proliferation was performed by enzymatic assay as described [26]. For apoptosis, caspase 3/7 activity was measured using the Apo-one Homogeneous Caspase-3/7 Assay kit (Promega, Madison, WI).

### Colony formation assay

Briefly, 6 well dishes were seeded with 500 viable cells and allowed to grow for 24 h. The cells were then incubated in the presence or absence of 30 µM curcumin for 24 h. Curcumin containing medium was then removed, and the cells were washed in PBS and incubated for an additional 10 d in complete medium. Each treatment was done in triplicate. The colonies obtained were washed with PBS and fixed in 10% formalin for 10 min at room temperature and then washed with PBS followed by staining with Crystal violet. The colonies were counted and compared with untreated cells.

### Cell cycle analyses

Cells were treated with 30 µM of curcumin for 12 and 24 h, then cells were trypsinized and suspended in phosphate buffered saline (PBS). Single-cell suspensions were fixed using 70% ethanol for 2 h, and subsequently permeabilized with PBS containing 1 mg/ml propidium iodide (Sigma-Aldrich), 0.1% Triton X-100 (Sigma-Aldrich) and 2 µg DNase-free RNase (Sigma-Aldrich) at room temperature. Flow cytometry was done with a FACSCalibur analyzer (Becton Dickinson, Mountain, View, CA), capturing 10,000 events for each sample. Results were analyzed with ModFit LT ™ software (Verity Software House, Topsham, ME).

### Real Time Reverse-Transcription Polymerase Chain Reaction Analysis

Total RNA isolated from TE-7 cells using TRIZOL reagent was reverse transcribed with Superscript II reverse transcriptase in the presence of random hexanucleotide primers (all from Invitrogen, Carlsbad, CA). Complementary DNAs were then used for Real Time PCR using Jumpstart Taq DNA polymerase (Sigma-Aldrich) and SYBR Green nucleic acid stain (Molecular Probes, Eugene, OR). Crossing threshold values for individual genes were normalized to β-Actin. Changes in mRNA expression were expressed as fold change relative to control. Primers used in this study were as follows: β-Actin: 5′-GCTGATCCACATCTGCTGG-3′ and 5′-ATCATTCTCCTCCTCAGCG-3′; Cyclin D1: 5′-AATGACCCCGCACGATTTC-3′ and 5′-TCAGGTTCAGGCCTTGCAC-3′; Notch-1: 5′-CACTGTGGGCGGGTCC-3′ and 5′-GTTGTATTGGTTCGGCACCAT-3′; Hes-1 5′-AGGCGGACATTCTGGAAATG-3′ and 5′-CGGTACTTCCCCAGCACACTT-3′; Presenilin-1 5′ ATCATGCTCTTTGTCC-3′ and 5′- TCTTCTGTGAATGGG-3′; Nicastrin 5′-CAGATTGGCTGCCAGT-3′ and 5′-CTCCAGCAGAACCAT-3′.

### miRNA analysis

Total miRNA was isolated using mirVana™ miRNA isolation kit (Ambion Inc, Grand Island, NY). Total miRNA isolated from TE-7 cells were subjected to reverse transcription with Superscript™ II RNase H-Reverse Transcriptase and random hexanucleotide primers (Invitrogen). The cDNA was subsequently used to perform Real-time PCR by SYBR chemistry (SYBR® Green I; Molecular Probes) for pri-*let-7a* transcript using specific primers and Jumpstart Taq DNA polymerase (Sigma-Aldrich, St. Louis, MO). The crossing threshold value assessed by Real time PCR was noted for *pri-let-7a* miRNA and normalized with *U6* pri-miRNA. The changes in pri-miRNA were expressed as fold change relative to control with ± SEM value. Primers used are: *pri-U6*: 5′-CTCGCTTCGGCAGCACA-3′ and 5′-AACGCTTCACGAATTTGCGT-3′, *pri-let-7a*: 5′-GAGGTAGTAGGTTGTATAGTTTAGAA-3′, 5′-AAAGCTAGGAGGCTGTACA-3′. Pri-miR-34 a 5′-TGGCAGTGTCTTAGCTGGTTG-3′ and 5′- GGCAGTATACTTGCTGATTGCTT-3′, pri-miR-21:5′-GCTTATCAGACTGATGTTGACTG-3′ and 5′-CAGCCCATCGACTGGTG-3′.

### Western Blot Analysis

Cell lysates were subjected to polyacrylamide gel electrophoresis and blotted onto Immobilon-P polyvinylidene difluoride membranes (Millipore, Bedford, MA). Notch-1, Caspase 3, Bcl2 and Bax antibodies were purchased from Cell Signaling Technology (Beverly, MA). Cyclin D1, Notch-1, Jagged-1, Hes-1 and Actin antibodies were purchased from Santa Cruz Biotechnology Inc (Santa Cruz, CA). Presenilin 1, 2, Nicastrin, APH1 and Pen2 antibodies from GenScript Inc (Piscataway, NJ) and specific proteins were detected by the enhanced chemiluminescence system (GE Healthcare, Piscataway, NJ).

### Spheroid assay

For formation of spheroids, cells were cultured in RPMI 1640 (Mediatech) supplemented with 20 ng/ml bFGF (Invitrogen) 10 mL per 500 mL of 50X B27 supplement (Invitrogen) EGF 20 ng/ml (Invitrogen) and antibiotic and antimycotic solution. Cells were seeded at low densities (5000 cells/mL) in 6 well low adhesion plates. The cells were treated with increasing concentrations of curcumin (0–50 µM). After 7 days the spheroids were photographed.

### Immunofluorescence staining

The cells were plated over night on cover-slips on six well plates. After treatment with 30 µM of curcumin for 24 h, cells were then fixed with paraformaldehyde for 15 min, rinsed with PBS, and incubated with 2% bovine serum albumin in PBS for 30 min. The cells were then incubated overnight with anti-Notch-1, anti-Jagged-1, anti-Hes-1, anti-Presenilin 1 and anti-Nicastrin antibody, respectively. After washing with PBS, the cells were incubated with Cy-3-conjugated secondary antibody for 30 min and washed with PBS. Cell images were observed under a fluorescent microscope.

### Statistical analysis

All values are expressed as the mean ± SEM. Data was analyzed using an unpaired 2-tailed t test. *P* value of less than 0.05 was considered statistically significant.

## Results

### Curcumin inhibits esophageal cancer cell growth

**Figure 1:**
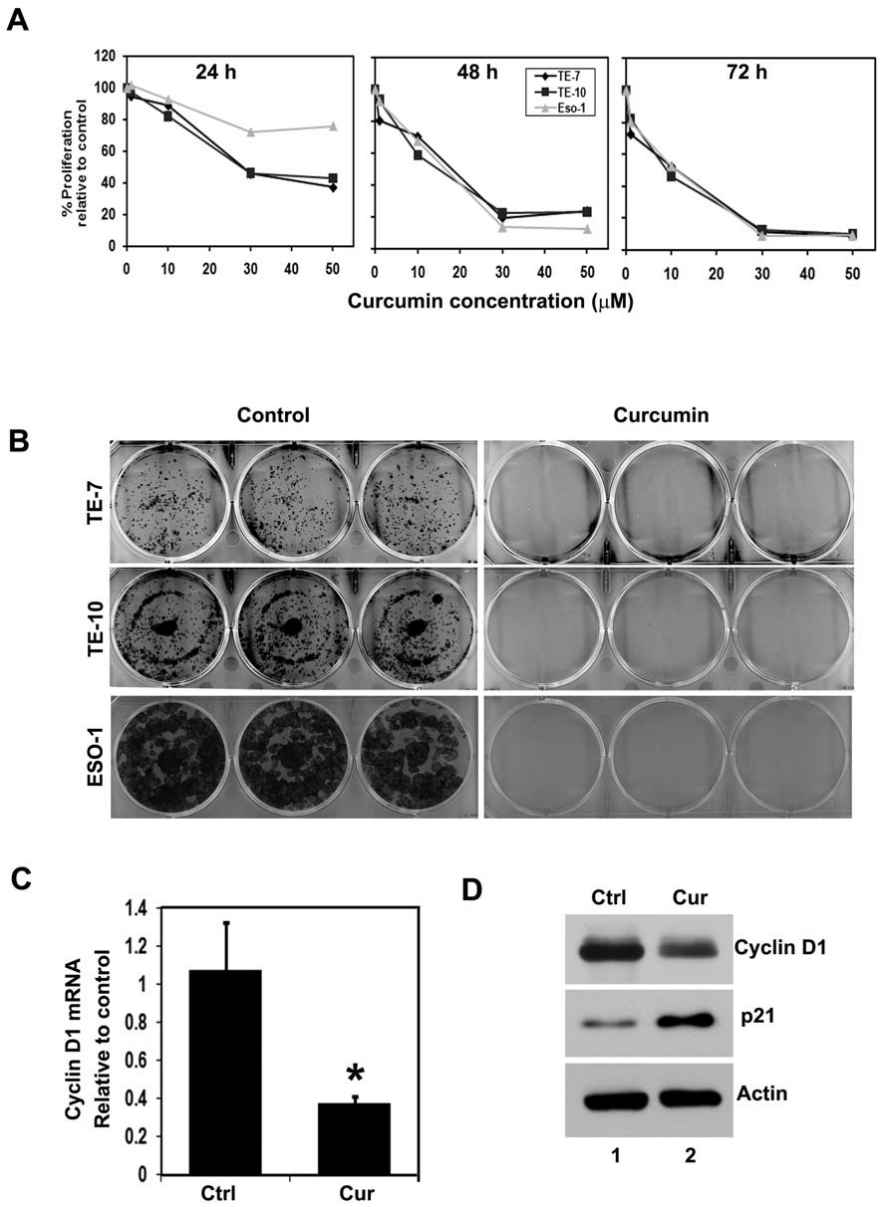
Curcumin inhibits esophageal cancer cell proliferation. (A) Curcumin inhibits proliferation of esophageal cancer cells. Cells were incubated with increasing doses of curcumin (0–50 µM) for up to 72 h and analyzed for cell proliferation. Curcumin treatment resulted in a significant dose- and time-dependent decrease in cell proliferation in all three cells when compared with untreated controls. (B) Curcumin inhibits colony formation. Esophageal cancer cells were incubated with 30 µM of curcumin for 24 h and allowed to grow into colonies for 10 days. Incubation with curcumin inhibits colony formation. Results are representative of three independent experiments. (C) Cyclin D1 is one the cell cycle regulatory protein and which is involved in the cell cycle arrest. RNA from TE-7 incubated with 30 µM curcumin was subjected to Real Time PCR for cyclin D1 mRNA expression. Curcumin treatment significantly inhibits cyclin D1 mRNA expression (*p<0.05). (D) Lysates from TE-7 incubated with 30 µM curcumin were analyzed by western blotting for cyclin D1 expression levels using mouse anti-cyclin D1 antibody. Curcumin treatment inhibits cyclin D1 protein expression.

We determined the effect of curcumin on esophageal cancer cell proliferation in a variety of cultured cell lines (TE-7, TE-10 and Eso-1). Curcumin significantly suppressed the proliferation of esophageal cancer cell lines TE-7, TE-10 and Eso-1 in dose and time dependent manner. This anti-proliferation effect on tumor cells was seen within a 24 h period, which continued to significantly increase over the next 72 h (Figure 1A). To determine the long-term effect of curcumin treatment, cells were treated with 30 µM curcumin for 24 h, following which the cells were allowed to grow in normal media. Curcumin treatment suppressed colony formation in all esophageal cancer cell lines (Figure 1B), suggesting that curcumin's effect on the tumor cells was irreversible. Cyclin D1 is one the cell cycle regulatory protein that regulates the G1 to S-phase transition of the cell cycle and functions as a cofactor for several transcription factors in numerous cell lines. This cyclin forms a complex with and functions as regulatory subunit of CDK4 or CDK6, whose activity is required for G1/S transition. Cyclin D1 overexpression has been linked to the development and progression of cancer [27]. Curcumin treatment inhibited cyclin D1 mRNA and protein expression suggesting that it inhibits cancer cell proliferation. On the other hand, expression of p21 protein was increased (Figure 1C and D).

### Curcumin induces cell death by apoptosis

**Figure 2:**
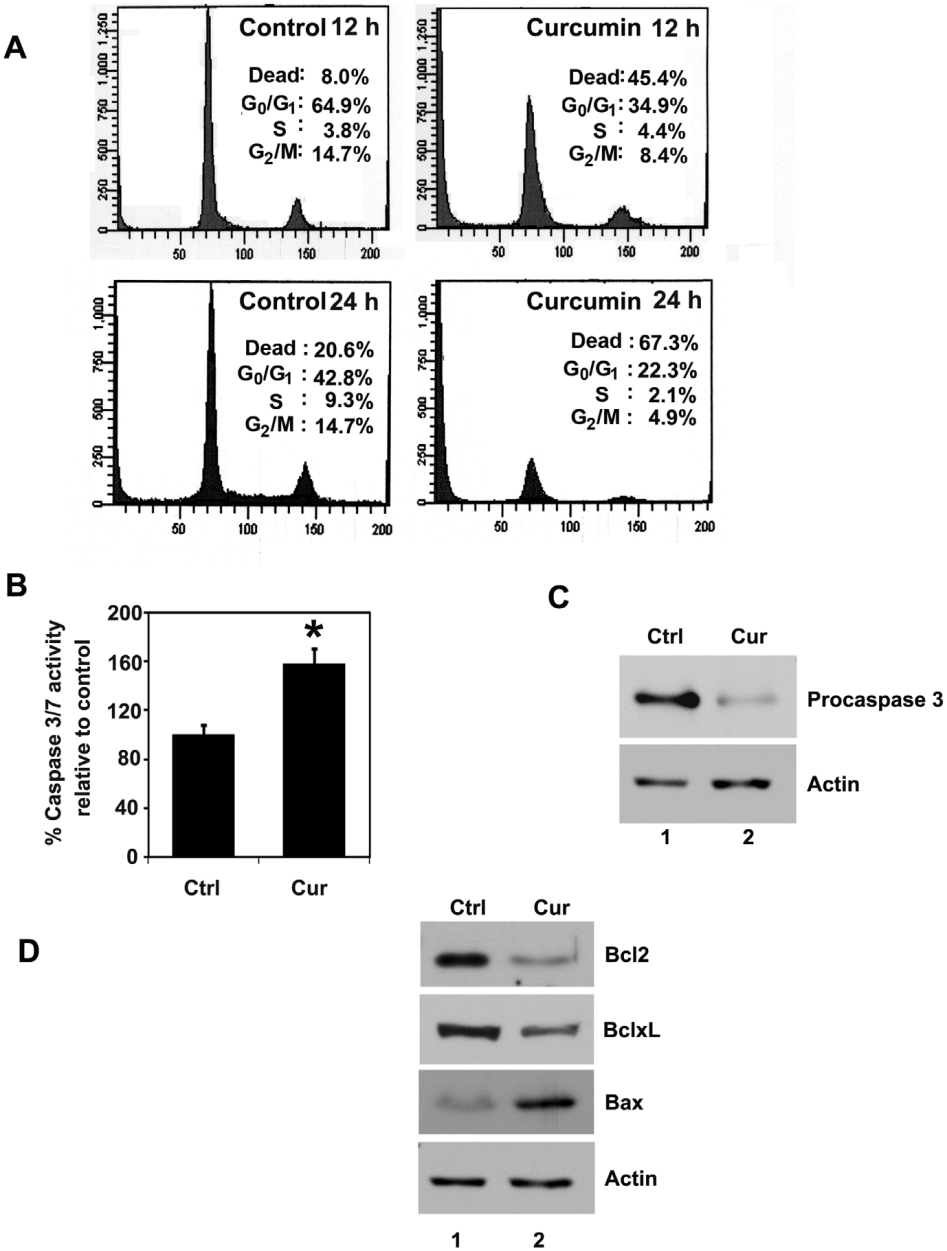
Curcumin induces cell death and apoptosis. (A) Cell cycle analysis of curcumin treated cells. TE-7 cells were treated with 30 µM of curcumin for 12 and 24 h, examined by flow cytometry following propidium iodide staining for DNA content. Curcumin treatment leads to increased number of dead cells. Graphs are representative of data collected from three experiments. (B) Curcumin induces caspase-3, an apoptosis mediator. TE-7 cells incubated with 30 µM of curcumin were analyzed for apoptosis by caspase-3 and-7 activation. Curcumin treatment increased the number of cells undergoing apoptosis compared to untreated controls (*p<0.05). (C) Lysates from TE-7 cells incubated with 30 µM of curcumin were analyzed by western blotting for caspase-3 protein levels using rabbit anti-caspase-3 antibody. Curcumin treatment resulted in decreased procaspase-3. (D) Lysates from TE-7 cells incubated with 30 µM of curcumin were analyzed by western blotting for Bcl2, BclxL, and Bax proteins. Curcumin reduces expression of anti-apoptotic proteins Bcl2 and BclxL, whereas increased expression of pro-apoptotic proteins in treated cells when compared to untreated cells.

Given the effects of curcumin on suppression of proliferation and colony formation, we next determined whether curcumin affects cell cycle progression. Treatment with curcumin significantly induced cell death of TE-7 cells within 12 h and continuously increased up to 24 h (Figure 2A). Such similar increases in cell death have been observed in other cell types [28]. Curcumin is known to induce apoptosis in variety of cancer cells. The caspases are a family of proteins known for the execution of apoptosis. Caspase-3 and caspase-7 are key effector molecules known to induce apoptosis in variety of cancer cells by amplifying the signal from initiator caspases, such as caspase-8 or caspase-10. Increased activation of effector caspase-3 and caspase-7 was observed within 24 hours in the TE-7 cell line treated with curcumin suggesting induction of apoptosis (Figure 2B). Western blot analyses of TE-7 cell lysates demonstrated a significant decrease in procaspase-3 in curcumin treated tumor cells (Figure 2C). Further confirmation that the cells were undergoing apoptosis was obtained by western blot analyses for anti-apoptotic Bcl2 and BclxL and pro-apoptotic Bax proteins. Curcumin treatment inhibited the expression of Bcl2 and BclxL and increased Bax protein levels. These data suggest that curcumin is a potent inducer of apoptosis in esophageal cancer cells (Figure 2D).

### Curcumin inhibits esophagosphere formation

**Figure 3:**
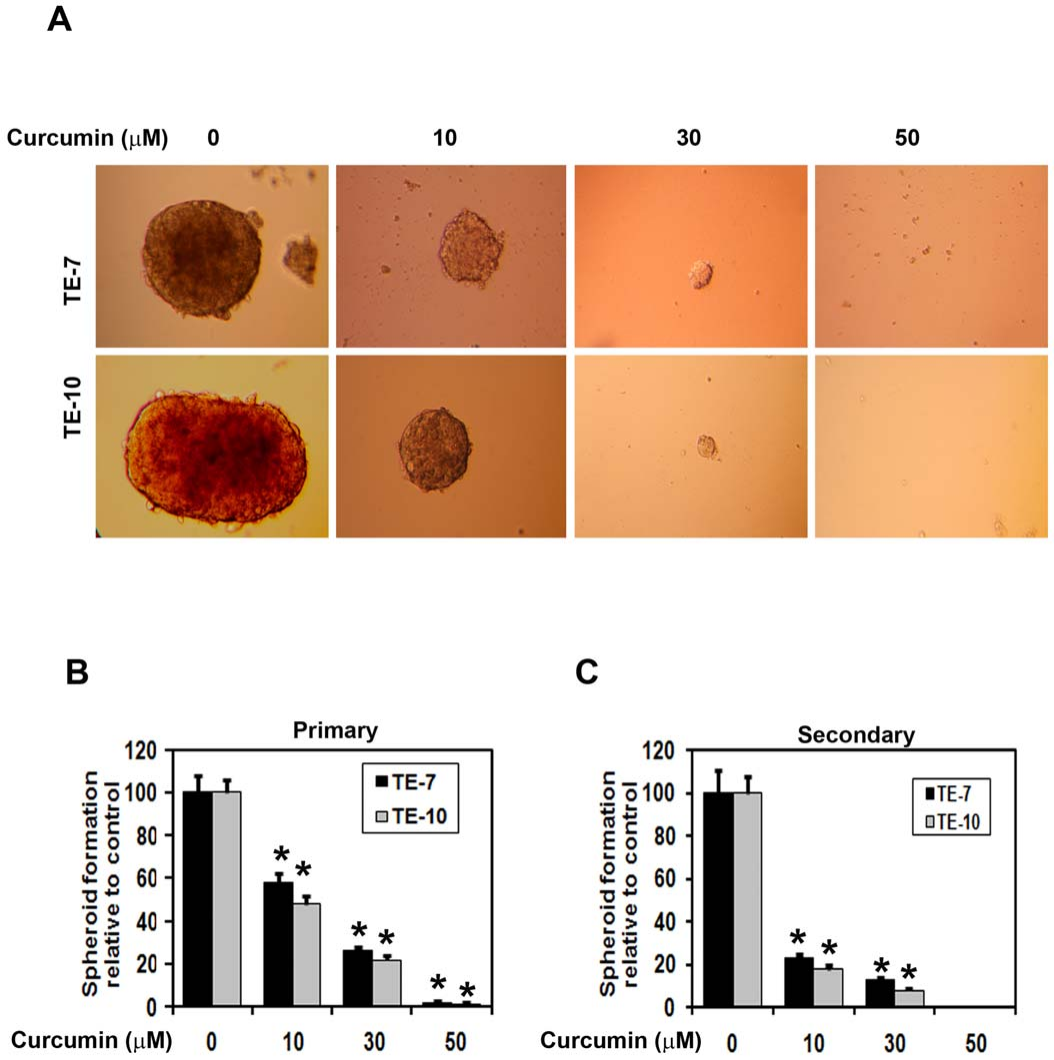
Curcumin treatment inhibits esophageal cancer cells spheroid formation. (A) TE-7 and TE cells were grown in low adherent plates and treated with increasing concentrations of curcumin (0–50 µM) and performed for the spheroid assay. After one week, the spheroids were photographed. (B) Spheroid was counted and performed bar diagram. Curcumin treatment significantly inhibited esophageal cancer cells spheroids (*p<0.05). (C) The primary spheroids were collected and separated into single cells and replated. Curcumin treatment significantly inhibited esophageal cancer cells secondary spheroids (*p<0.05).

Given that curcumin inhibits colonosphere formation in colon cancer cells and affecting Notch signaling pathway proteins. We next determined the effects of curcumin on esophageal cancer cell spheroid formation. Curcumin treatment significantly inhibited esophagosphere formation in a dose dependent manner in both TE-7 and TE-10 cells (Figure 3A). The spheroids size, numbers were counted. Curcumin treatment reduced both primary and secondary spheroid formation (Figure 3B and C).

### Curcumin inhibits Notch activation by downregulating the γ-secretase complex

**Figure 4:**
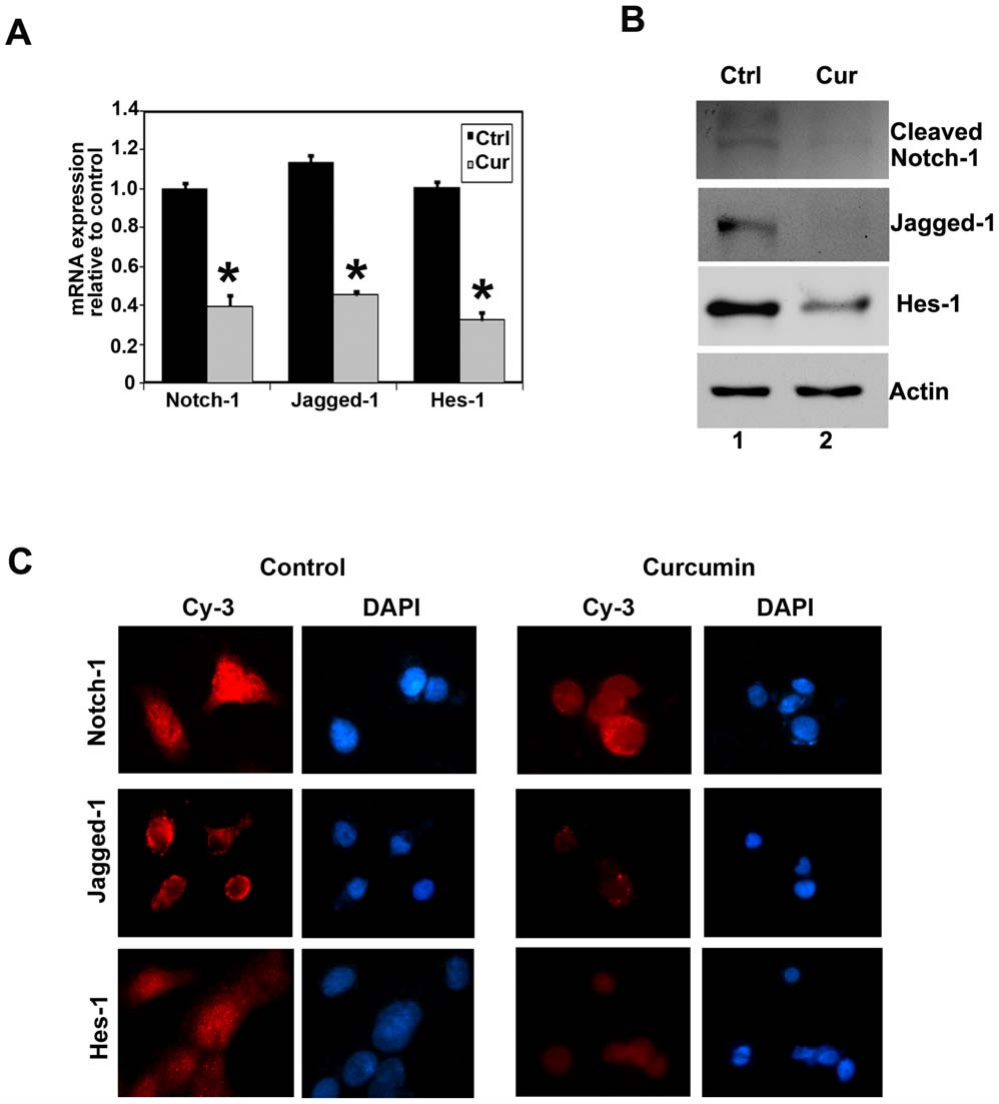
Curcumin inhibits notch signaling and its downstream targets proteins. (A) real-time reverse transcription-PCR analysis of total RNA from TE-7 cells following 30 µM of curcumin treatment for 24 h showed reduction in the expression of Notch-1, its ligand Jagged-1 and its target gene Hes-1 mRNA (*p<0.05). (B) lysates from curcumin treatment caused significant reduction in the expression of cleaved Notch-1, its ligand Jagged-1and its target gene Hes-1 protein levels in TE-7 cells. (C) TE-7 cells treated with 30 µM of curcumin for 24 h were subjected to immuno-fluorescent staining using anti-Notch-1, anti-Jagged-1 and anti-Hes-1 antibodies. Curcumin treatment resulted in lower levels of Notch-1 protein in the nucleus and reduced Jagged-1 and Hes-1 expression in TE-7 cells.

Notch-1 is a cell membrane associated protein. Ligand engagement causes the intracellular domain of the transmembrane receptor Notch (NICD) to be cleaved from the membrane through the action of the γ-secretase complex. NICD translocates to the nucleus, where it associates with a family of DNA binding proteins to activate transcription of Notch target genes such as hairy and enhancer-of-split 1 (*Hes1*) [29]. We determined the effect of curcumin on Notch-1, its ligand Jagged-1 and Hes-1 in TE-7 cells. Curcumin treatment significantly downregulated Notch-1 and its ligand Jagged-1 and downstream target proteins Hes-1 both mRNA and protein levels (Figure 4A and B). Protein levels were confirmed by immune-fluorescence staining, where significantly lower levels of nuclear Notch-1 and cytoplasmic Jagged-1 were observed in the curcumin treated cells (Figure 4C).

**Figure 5:**
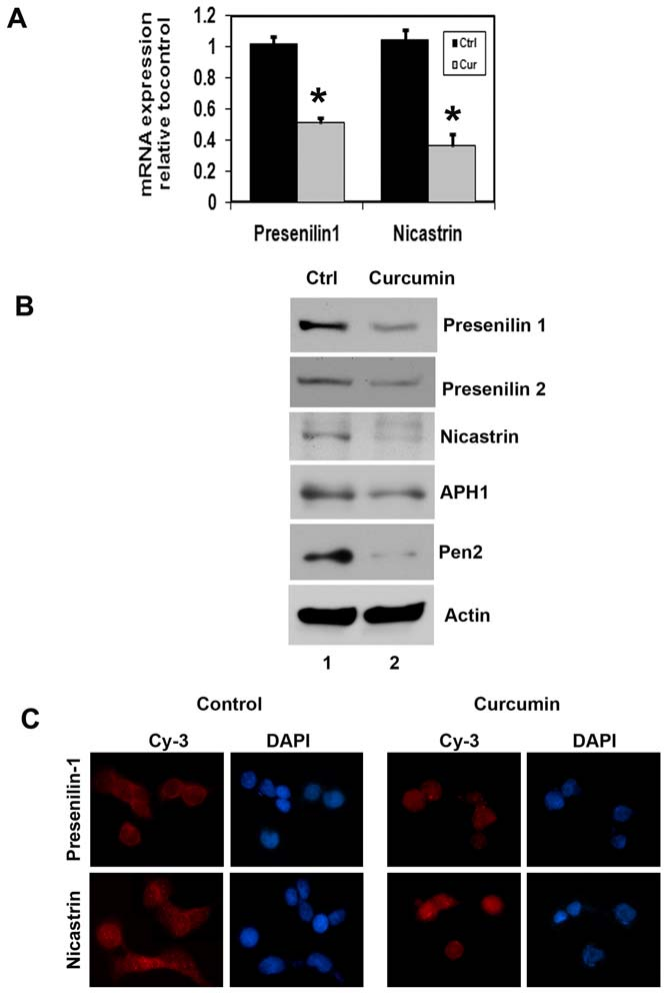
Curcumin inhibits γ-secretase complex proteins. (A) real-time reverse transcription-PCR analysis of total RNA from TE-7 cells following 30 µM of curcumin treatment for 24 h showed reduction in the expression of Presenilin 1 and Nicastrin mRNA (*p<0.05). (B) lysates from curcumin treatment caused significant reduction in the expression of γ-secretase complex proteins Presenilin 1 and 2, Nicastrin, APH1 and Pen2 protein levels in TE-7 cells. (C) TE-7 cells treated with 30 µM of curcumin for 24 h were subjected to immuno-fluorescent staining using anti-Presenilin 1 and anti-Nicastrin antibodies. Curcumin treatment resulted in lower levels of Presenilin 1 and Nicastrin expression in TE-7cells.

We next determined the mechanism by which curcumin affects Notch-1 activation. γ-secretase is a multi-protein complex containing an intra-membrane cleaving protease. The complex has a growing list of proteins substrates, including the Notch receptors. The four components of γ-secretase complex, Presenilin 1, Nicastrin, Pen2, and Aph1 are all thought to be essential for activity [13] [30] [31]. The catalytic domain resides within Presenilin, while Nicastrin has been suggested to be critical for substrate recognition [32]. Curcumin treatment resulted in down-regulation in the expression of γ-secretase complex proteins, Presenilin 1 and 2, Nicastrin, APH1 and PEN2 (Figure 5A, B and C). These data suggest that curcumin mediated down-regulation of the Notch signaling pathway occurs in part through the inhibition of the γ-secretase complex.

### Combination of curcumin and a γ-secretase complex inhibitor DAPT further affects esophageal cancer growth

**Figure 6:**
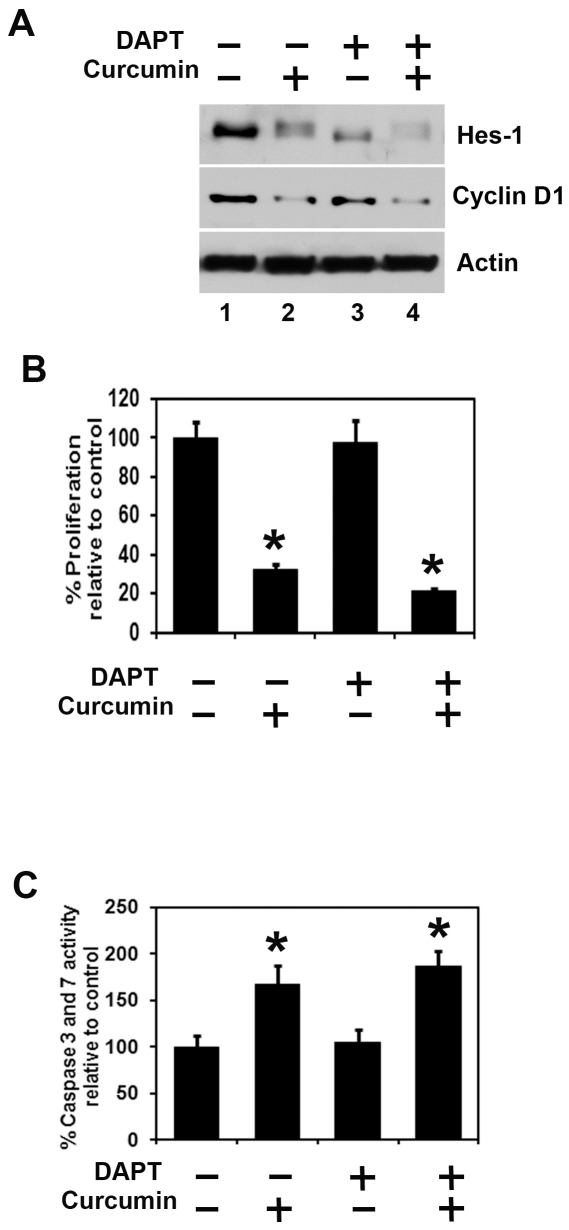
Combination of Curcumin and DAPT further inhibit proliferation and induce apoptosis. (A) TE cells treated with DAPT (50 µM) and curcumin (30 µM) alone and in combination for 24 h. Lysates were analyzed by western blotting. Hes-1 and Cyclin D1 proteins were further decreased with the combination of the two compounds. (B) TE cells treated with DAPT (50 µM) and curcumin (30 µM) alone and in combination for 48 h. Cell proliferation was significantly inhibited following treatment with the combination of DAPT and curcumin when compared to each curcumin alone using hexosaminidase enzyme assay (*P<0.05). (C) Apoptosis was significantly induced following treatment with the combination of DAPT and curcumin when compared to each curcumin alone using Apo-one Homogeneous Caspase-3/7 Assay kit (*P<0.05).

Treatment with combination of curcumin and a γ-secretase complex inhibitor (GSI) DAPT further inhibits Notch-1 target proteins Hes-1 and cyclin D1 expression (Figure 6A). We performed combination of curcumin with a γ-secretase complex inhibitor DAPT on proliferation and apoptosis. Combination of curcumin and DAPT treatment further inhibits proliferation and induces apoptosis in TE-7 cells (Figure 6B and C). Similar results were observed in TE-10 cells (data not shown).

### Curcumin inhibits oncomir miRNA and increases tumor suppressor miRNA in esophageal cancer cells

**Figure 7:**
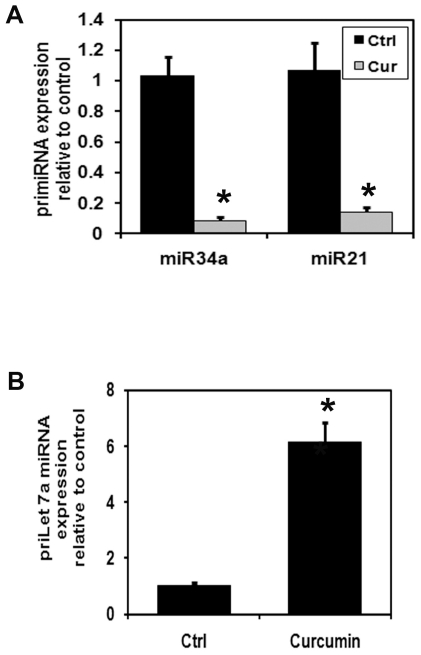
Curcumin treatment inhibits oncomiR miRNA in esophageal cancer cells. (A) real-time reverse transcription-PCR analysis of total miRNA from TE-7 cells following 30 µM of curcumin treatment for 24 h. Curcumin treatment significantly inhibit oncomiR miRNA expression in TE-7 cells (*P<0.05). (B) Curcumin treatment significantly up-regulated tumor suppressor let-7a miRNA expression in TE-7 cells.

Curcumin alters microRNA expression in colon [33] pancreas [34], breast [35], bladder [36] and lung [37] cancer cells. Recently, we have demonstrated that miRNAs as biomarkers for Barrett's esophagus progression and miR-21 is upregulated 12.57 fold in esophageal cancer [21]. We next determined the effects of curcumin on oncomir miRNA and tumor suppressor miRNA esophageal cancer cells. Curcumin treatment significantly down regulated miR-21 and miR-34a expression while upregulating let-7a miRNA expression (Figure 7A and B).

## Discussion

Esophageal cancer is one of the sixth leading causes of cancer-related deaths in the western world. Overall incidence of the disease is highest in men over 50 years of age [2]. Esophageal cancer is generally diagnosed at a late stage and has a poor prognosis, with a 5-year survival of less than 10%. The significant morbidity, toxicity and poor response rates of current chemotherapy regimens have led to searches for less toxic alternative therapies. We and others have shown that curcumin suppresses proliferation and induces apoptosis in a variety of tumor cells, including pancreas, breast, colon, oral, lung, melanoma, myeloma, leukemia, and prostate carcinoma. Previous studies have shown that curcumin suppresses the proliferation and increases apoptosis in esophageal cancer cells [5] [6] [38] [39].

**Figure 8:**
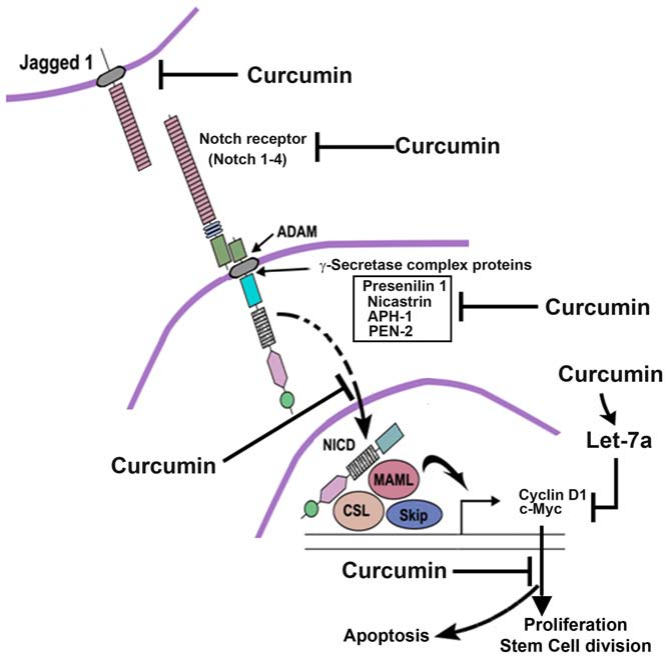
Schematic diagram of the effect of curcumin on Notch signaling in esophageal cancer. Our studies demonstrate that curcumin inhibits the expression of Jagged-1 and the Notch-1 receptor. Curcumin also inhibits γ-secretase complex proteins, thereby inhibiting cleavage of the Notch receptor. As a result, the Notch intracellular domain (NICD) is not released and therefore does not translocate to the nucleus to activate the downstream target genes c-myc and cyclin D1. This results in inhibition of cell proliferation and of stem cell regeneration, while at the same time the induction of apoptosis.

The data presented in this article show that curcumin selectively inhibits the proliferation of esophageal cancer cells, suppresses the formation of esophageal cancer cell colonies, promotes cell cycle arrest and apoptosis, inhibits esophagosphere formation, down regulates notch signaling and its γ-secretase complex proteins, and also inhibits oncomir miRNA expression and induces tumor suppressor miRNA expression. These results are further presented in the schematic diagram in Figure 8. Our results show that curcumin can effectively suppress cell proliferation within 24 h. Moreover, the inhibitory effects on tumor cells appear to be sustained and irreversible after 24 h of treatment. Our data with cyclin D1 is intriguing in that there was a reduction in its expression at 24 h. This should have resulted in cells undergoing G0-G1 arrest since the protein is responsible for progression through the G0–G1/S phase transition [40]. Similar curcumin-induced G0/G1 arrest has also been shown to occur in other cancer cell types, including colon and gastric cancers, and in mantle cell lymphoma [28] [41]. Indeed, we observed a significant increase in dead cells in the esophageal cancer cells even at 12 h following incubation with curcumin, which subsequently led to cell death.

In the present study, we also found that curcumin down regulates Notch signaling through the inhibition of the γ-secretase complex. Curcumin inhibits Notch-1 and its ligand Jagged-1 in esophageal cancer cells. We also found that curcumin inhibited the expression of the Notch-1 downstream target *Hes-1*. Recently, it has been reported that the Notch pathway plays critical roles in the processes of tumor cell proliferation, apoptosis and stem cell maintenance and is closely associated with tumorigenesis in esophageal cancer cells [42]. Therefore, curcumin mediated cell growth inhibition could be partly mediated via inactivation of Notch-1 activity. This was further confirmed by the combination of a GSI and curcumin, which further inhibited proliferation and induced apoptosis. Similarly, the combination of curcumin with a GSI further inhibited Hes-1 and cyclin D1 expressions. However, Notch-1 is not the only pathway active in esophageal cancer as many other cellular pathways are activated [7] [39]. It would be interesting to determine whether curcumin is equally potent in inhibiting other signal transduction pathways. In addition, it would be interesting to determine whether ectopic expression of Notch downstream target genes such as Hes-1 and c-myc in the condition of Notch knockdown or rescue protects from curcumin-mediated cell death.

Notch signaling is required for stem cell self-renewal and maintaining the stemness [43]. One method that is commonly used to demonstrate stemness is the growth of spheroids in ultra-low binding tissue culture dishes. 3D-spheroid culture allows for early stemness preservation. For esophageal-derived cells, these spheroids are called esophagospheres. Curcumin is a potent inhibitor of esophagosphere formation suggesting that it targets Notch signaling to affect cancer stem cells.

MicroRNAs can normalize multiple coding genes associated with tumor growth, and thus, assessment of specific miRNA expression could be useful for predicting disease outcome. It is well known that the development of cancer involves alterations in the expression of multiple genes regulated by transcriptional, post-transcription, translational, and posttranslational modification, and thus, a single gene or protein expression cannot accurately reflect the status of the disease. We have recently demonstrated that miRNAs [18] as biomarkers for Barrett's esophagus progression [21]. Several miRNAs upregulated significantly and specifically miR-21 is up-regulated 12.57 fold in esophageal cancer [21]. miR-21 is overexpressed in many solid tumors and has been shown to be associated with tumor progression, poor survival, and reduced therapeutic effects [44]. Curcumin treatment significantly reduced miR-21 expression in colon and pancreatic cancers, thereby inhibiting invasion and metastasis in colon cancer [33] [45]. Our result in esophageal cancer cells correlates with these previous findings. Several targets have been identified for miR-21. The microRNA has been shown to downregulate expression of tumor suppressor gene Pdcd4 in colorectal cancer cells, thereby stimulating tumor invasion, intravasation and metastasis [18]. miR-21 can also inhibit apoptosis in breast cancer cells by regulating Bcl2 expression [46]. Finally, miR-21 can inhibit gemcitabine-induced apoptosis via the PTEN and PI-3 kinase pathway [20]. It would be interesting to determine whether curcumin affects the expression of these genes in the esophageal cancer cells, and whether this expression can be modulated by miR-21.

In our current study we did not observe increased miR-34a expression in response to curcumin, which was surprising because miR-34a is a tumor suppressor microRNA and upregulation of this microRNA induces cells to undergo apoptosis. However, previous studies have demonstrated that the altered expression of miR-34a in esophageal cancer tissue specimens did not show a significant association with the patients' clinico-pathologic parameters and survival [47]. Also, tumor suppressor protein p53 is known to induce miR-34a expression thereby leading cells to apoptosis. However, esophageal cancer cells (and cell lines) are either p53 deficient (TE-7) or have mutations leading to accumulation of mutant, non-functional p53 protein (TE-10) [48]. This could be a reason why miR-34a is not upregulated in response to curcumin treatment. One other reason for the lack of miR-34a upregulation could be that previous studies have shown that in cells where myc protein is upregulated, miR-34a will inhibit p53-dependent apoptosis [49]. c-myc is upregulated in 90% of esophageal adenocarcinomas, and in esophageal cancer cells [50]. Hence, the possibility exists that curcumin circumvents the negative effect of c-myc by not upregulating miR-34a.

Let-7a is a tumor suppressor miRNA and is down regulated in many cancers. Curcumin treatment significantly increased let-7a miRNA expression. A recent study demonstrated that let-7a inhibits proliferation of human prostate cancer cells by targeting E2F2 and CCND2. Both these proteins are cell-cycle regulators and their aberrant expression can lead to abnormal cellular proliferation [51]. There is also an interesting link between curcumin, microRNAs and Notch-1 regulation. Curcumin upregulates the expression of miR-22 in human pancreatic cancer cells especially [34]. Recent studies have also shown that treatment of PanC-1 or Colo-357 cells with genistein (isoflavone) showed decreased expression of the oncogenic miRNA such as miR-17, miR-20a, miR-106a, and increased the expression of the tumor suppressor miRNAs such as let-7, miR-16-1 [52]. More importantly, a recent study by Sarkar and colleagues demonstrated that Notch-1 induced the expression of miR-21 while inhibiting the expression of let-7a [53]. Our studies fit with these results in that while curcumin inhibited Notch-1 expression, it also inhibited expression of miR-21 while inducing let-7a.

In conclusion, these studies provide mechanistic evidence that curcumin treatment inhibits cell growth *in vitro*. Curcumin seems to have multiple molecular targets and its enhanced potency in cancer in various cancer cell lines and xenograft tumors renders it a strong candidate for therapeutic applications for esophageal cancer as well as other cancers and inflammatory disease states.

## References

[1] Dawsey SP, Tonui S, Parker RK, Fitzwater JW, Dawsey SM (2010). Esophageal cancer in young people: a case series of 109 cases and review of the literature.. PLoS One.

[2] Bosetti C, Levi F, Ferlay J, Garavello W, Lucchini F (2008). Trends in oesophageal cancer incidence and mortality in Europe.. Int J Cancer.

[3] Siegel R, Ward E, Brawley O, Jemal A (2011). Cancer statistics, 2011: the impact of eliminating socioeconomic and racial disparities on premature cancer deaths.. CA Cancer J Clin.

[4] Liao S, Xia J, Chen Z, Zhang S, Ahmad A (2011). Inhibitory effect of curcumin on oral carcinoma CAL-27 cells via suppression of Notch-1 and NF-kappaB signaling pathways.. J Cell Biochem.

[5] O'Sullivan-Coyne G, O'Sullivan GC, O'Donovan TR, Piwocka K, McKenna SL (2009). Curcumin induces apoptosis-independent death in oesophageal cancer cells.. Br J Cancer.

[6] Hartojo W, Silvers AL, Thomas DG, Seder CW, Lin L (2010). Curcumin promotes apoptosis, increases chemosensitivity, and inhibits nuclear factor kappaB in esophageal adenocarcinoma.. Transl Oncol.

[7] Tian F, Song M, Xu PR, Liu HT, Xue LX (2008). [Curcumin promotes apoptosis of esophageal squamous carcinoma cell lines through inhibition of NF-kappaB signaling pathway].. Ai Zheng.

[8] Androutsellis-Theotokis A, Leker RR, Soldner F, Hoeppner DJ, Ravin R (2006). Notch signalling regulates stem cell numbers in vitro and in vivo.. Nature.

[9] Peters JH, Avisar N (2010). The molecular pathogenesis of Barrett's esophagus: common signaling pathways in embryogenesis metaplasia and neoplasia.. J Gastrointest Surg.

[10] Masuda S (2011). Dysfunctional transforming growth factor-beta signaling with constitutively active notch signaling in Barrett's esophageal adenocarcinoma.. Cancer.

[11] Miele L (2006). Notch signaling.. Clin Cancer Res.

[12] Miele L, Golde T, Osborne B (2006). Notch signaling in cancer.. Curr Mol Med.

[13] Miele L, Miao H, Nickoloff BJ (2006). NOTCH signaling as a novel cancer therapeutic target.. Curr Cancer Drug Targets.

[14] Katoh M (2007). Notch signaling in gastrointestinal tract (review).. Int J Oncol.

[15] Yao J, Duan L, Fan M, Wu X (2007). Gamma-secretase inhibitors exerts antitumor activity via down-regulation of Notch and Nuclear factor kappa B in human tongue carcinoma cells.. Oral Dis.

[16] Denli AM, Tops BB, Plasterk RH, Ketting RF, Hannon GJ (2004). Processing of primary microRNAs by the Microprocessor complex.. Nature.

[17] Esquela-Kerscher A, Slack FJ (2006). Oncomirs - microRNAs with a role in cancer.. Nat Rev Cancer.

[18] Asangani IA, Rasheed SA, Nikolova DA, Leupold JH, Colburn NH (2008). MicroRNA-21 (miR-21) post-transcriptionally downregulates tumor suppressor Pdcd4 and stimulates invasion, intravasation and metastasis in colorectal cancer.. Oncogene.

[19] Roldo C, Missiaglia E, Hagan JP, Falconi M, Capelli P (2006). MicroRNA expression abnormalities in pancreatic endocrine and acinar tumors are associated with distinctive pathologic features and clinical behavior.. J Clin Oncol.

[20] Meng F, Henson R, Wehbe-Janek H, Ghoshal K, Jacob ST (2007). MicroRNA-21 regulates expression of the PTEN tumor suppressor gene in human hepatocellular cancer.. Gastroenterology.

[21] Bansal A, Lee IH, Hong X, Anand V, Mathur SC (2011). Feasibility of mcroRNAs as biomarkers for Barrett's Esophagus progression: a pilot cross-sectional, phase 2 biomarker study.. Am J Gastroenterol.

[22] Hermeking H (2010). The miR-34 family in cancer and apoptosis.. Cell Death Differ.

[23] Wang Z, Li Y, Kong D, Ahmad A, Banerjee S (2010). Cross-talk between miRNA and Notch signaling pathways in tumor development and progression.. Cancer Lett.

[24] Li Y, Guessous F, Zhang Y, Dipierro C, Kefas B (2009). MicroRNA-34a inhibits glioblastoma growth by targeting multiple oncogenes.. Cancer Res.

[25] Lee ST, Chu K, Oh HJ, Im WS, Lim JY (2011). Let-7 microRNA inhibits the proliferation of human glioblastoma cells.. J Neurooncol.

[26] Landegren U (1984). Measurement of cell numbers by means of the endogenous enzyme hexosaminidase. Applications to detection of lymphokines and cell surface antigens.. J Immunol Methods.

[27] Alao JP (2007). The regulation of cyclin D1 degradation: roles in cancer development and the potential for therapeutic invention.. Mol Cancer.

[28] Su CC, Lin JG, Li TM, Chung JG, Yang JS (2006). Curcumin-induced apoptosis of human colon cancer colo 205 cells through the production of ROS, Ca2+ and the activation of caspase-3.. Anticancer Res.

[29] Wang Z, Azmi AS, Ahmad A, Banerjee S, Wang S (2009). TW-37, a small-molecule inhibitor of Bcl-2, inhibits cell growth and induces apoptosis in pancreatic cancer: involvement of Notch-1 signaling pathway.. Cancer Res.

[30] Li H, Wolfe MS, Selkoe DJ (2009). Toward structural elucidation of the gamma-secretase complex.. Structure.

[31] Wolfe MS (2009). gamma-Secretase in biology and medicine.. Semin Cell Dev Biol.

[32] Gong P, Vetrivel KS, Nguyen PD, Meckler X, Cheng H (2010). Mutation analysis of the presenilin 1 N-terminal domain reveals a broad spectrum of gamma-secretase activity toward amyloid precursor protein and other substrates.. J Biol Chem.

[33] Mudduluru G, George-William JN, Muppala S, Asangani IA, Kumarswamy R (2011). Curcumin regulates miR-21 expression and inhibits invasion and metastasis in colorectal cancer.. Biosci Rep.

[34] Sun M, Estrov Z, Ji Y, Coombes KR, Harris DH (2008). Curcumin (diferuloylmethane) alters the expression profiles of microRNAs in human pancreatic cancer cells.. Mol Cancer Ther.

[35] Yang J, Cao Y, Sun J, Zhang Y (2010). Curcumin reduces the expression of Bcl-2 by upregulating miR-15a and miR-16 in MCF-7 cells.. Med Oncol.

[36] Saini S, Arora S, Majid S, Shahryari V, Chen Y (2011). Curcumin modulates microRNA-203 mediated regulation of the Src-Akt axis in bladder cancer.. Cancer Prev Res (Phila).

[37] Zhang J, Du Y, Wu C, Ren X, Ti X (2010). Curcumin promotes apoptosis in human lung adenocarcinoma cells through miR-186* signaling pathway.. Oncol Rep.

[38] Ushida J, Sugie S, Kawabata K, Pham QV, Tanaka T (2000). Chemopreventive effect of curcumin on N-nitrosomethylbenzylamine-induced esophageal carcinogenesis in rats.. Jpn J Cancer Res.

[39] Yu L, Wu WK, Li ZJ, Wong HP, Tai EK (2008). E series of prostaglandin receptor 2-mediated activation of extracellular signal-regulated kinase/activator protein-1 signaling is required for the mitogenic action of prostaglandin E2 in esophageal squamous-cell carcinoma.. J Pharmacol Exp Ther.

[40] Ashworth T, Roy AL (2009). Phase specific functions of the transcription factor TFII-I during cell cycle.. Cell Cycle.

[41] Shishodia S, Amin HM, Lai R, Aggarwal BB (2005). Curcumin (diferuloylmethane) inhibits constitutive NF-kappaB activation, induces G1/S arrest, suppresses proliferation, and induces apoptosis in mantle cell lymphoma.. Biochem Pharmacol.

[42] Mendelson J, Song S, Li Y, Maru DM, Mishra B (2011). Dysfunctional transforming growth factor-beta signaling with constitutively active Notch signaling in Barrett's esophageal adenocarcinoma.. Cancer.

[43] Woo SM, Kim J, Han HW, Chae JI, Son MY (2009). Notch signaling is required for maintaining stem-cell features of neuroprogenitor cells derived from human embryonic stem cells.. BMC Neurosci.

[44] Schetter AJ, Leung SY, Sohn JJ, Zanetti KA, Bowman ED (2008). MicroRNA expression profiles associated with prognosis and therapeutic outcome in colon adenocarcinoma.. JAMA.

[45] Ali S, Ahmad A, Banerjee S, Padhye S, Dominiak K (2010). Gemcitabine sensitivity can be induced in pancreatic cancer cells through modulation of miR-200 and miR-21 expression by curcumin or its analogue CDF.. Cancer Res.

[46] Si ML, Zhu S, Wu H, Lu Z, Wu F (2007). miR-21-mediated tumor growth.. Oncogene.

[47] Hu Y, Correa AM, Hoque A, Guan B, Ye F (2011). Prognostic significance of differentially expressed miRNAs in esophageal cancer.. Int J Cancer.

[48] Barnas C, Martel-Planche G, Furukawa Y, Hollstein M, Montesano R (1997). Inactivation of the p53 protein in cell lines derived from human esophageal cancers.. Int J Cancer.

[49] Sotillo E, Laver T, Mellert H, Schelter JM, Cleary MA (2011). Myc overexpression brings out unexpected antiapoptotic effects of miR-34a.. Oncogene.

[50] Tselepis C, Morris CD, Wakelin D, Hardy R, Perry I (2003). Upregulation of the oncogene c-myc in Barrett's adenocarcinoma: induction of c-myc by acidified bile acid in vitro.. Gut.

[51] Dong Q, Meng P, Wang T, Qin W, Qin W (2010). MicroRNA let-7a inhibits proliferation of human prostate cancer cells in vitro and in vivo by targeting E2F2 and CCND2.. PLoS One.

[52] Vandenboom TG, Li Y, Philip PA, Sarkar FH (2008). MicroRNA and Cancer: Tiny Molecules with Major Implications.. Curr Genomics.

[53] Bao B, Wang Z, Ali S, Kong D, Li Y (2011). Notch-1 induces epithelial-mesenchymal transition consistent with cancer stem cell phenotype in pancreatic cancer cells.. Cancer Lett.

